# A Case of Abdominal Incisional Bladder Hernia

**DOI:** 10.7759/cureus.58955

**Published:** 2024-04-24

**Authors:** Atomu Suzuki, Michinari Suzuki, Satoshi Matsukuma, Kazuhisa Tokunou, Toru Kawaoka

**Affiliations:** 1 Department of Gastroenterological, Breast and Endocrine Surgery, JCHO Tokuyama Central Hospital, Shunan, JPN; 2 Department of Gastroenterological, Breast and Endocrine Surgery, Shunan City Shinnanyo Hospital, Shunan, JPN

**Keywords:** direct suture, hernia, abdominal incisional hernia, bladder hernia, open incisional hernia repair

## Abstract

The case is a woman in her 60s. She had been aware of lower abdominal distention and pain for six months but was under observation. Gradually, the patient experienced worsening pain during distention and became aware of distention, especially before urination. She visited our clinic. Ultrasound (US) and computed tomography (CT) revealed an abdominal incisional hernia. The hernia was in the bladder. We decided on surgical treatment and made a skin incision of about 3 cm just above the hernia portal. Since the size of the hernia portal was approximately 1.3 cm, the patient underwent direct suture closure to repair the hernia portal, and the surgery was completed. The postoperative course was good. The patient was discharged on the second postoperative day. Four months have passed since the surgery, and the patient is under observation without recurrence.

## Introduction

An abdominal incisional hernia is a postoperative complication that occurs in 3-11% of abdominal surgeries [1]. The small intestine is the most common hernia content [2]. Rarely, the bladder may prolapse. Most bladder hernias occur in the inguinal or femoral region, and abdominal incisional bladder hernias are extremely rare, especially after abdominal surgery. We present a case of an incisional bladder hernia with a literature review.

## Case presentation

The case is a woman in her 60s. The patient had been aware of lower abdominal distension and pain for six months but was being monitored. Gradually, the pain during distention worsened. She was seen in our clinic because she became aware of her symptoms, especially before urinating.

The performance status is 1, height is 160 cm, weight is 52 kg, and BMI is 20.3. The abdomen was flat and soft. No tenderness was noted in the abdomen. The cesarean section scar was located horizontally above the pubic bone. A bulge the size of half a ping-pong ball was observed in the same area, closer to the left side. It was easy to reverse the hernia.

**Figure 1:**
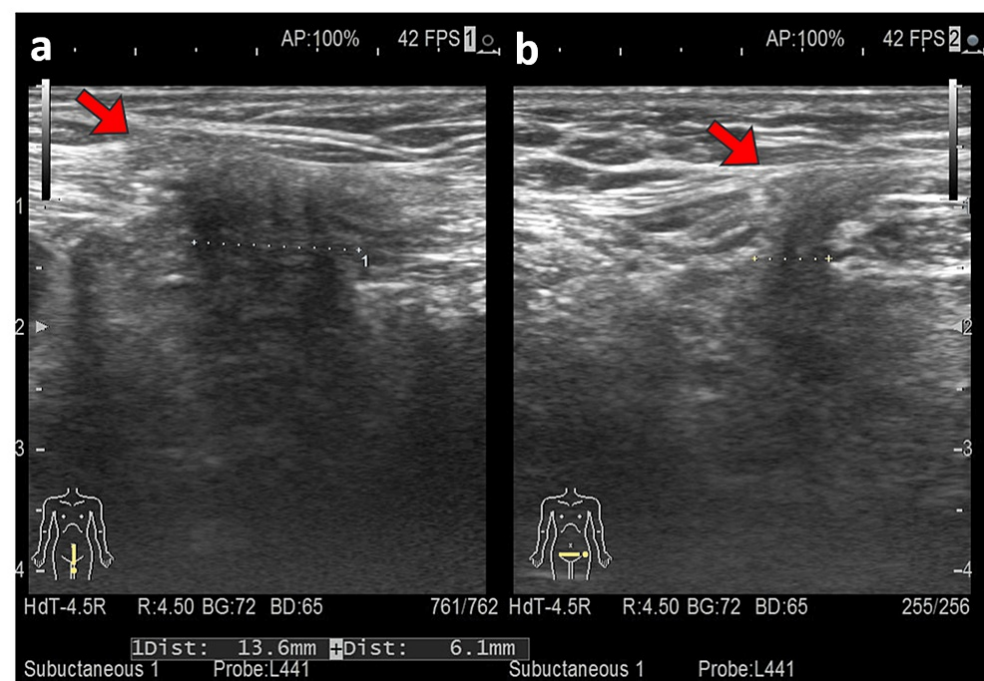
Ultrasonography revealing the presence of a hernia. The abdominal incisional hernia was observed. The hernia portal was about 1.3 cm × 6.1 mm.

The abdominal US and CT showed an abdominal incisional hernia in the left lower abdomen (hernia portal was about 1.3 cm) and a prolapsed bladder from the same area (Figure 1).

**Figure 2:**
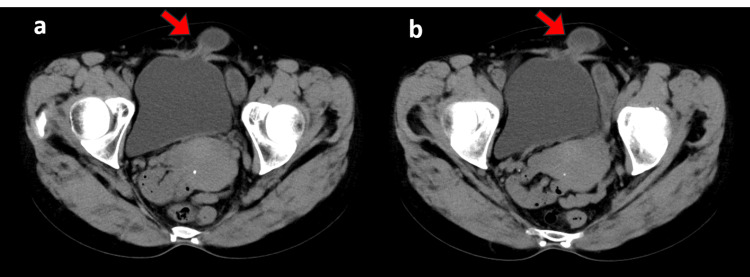
CT scan of the abdomen (axial). a, b: The abdominal incisional hernia in the left lower abdomen and a prolapsed bladder from the same area. The hernia portal was about 1.3 cm.

**Figure 3:**
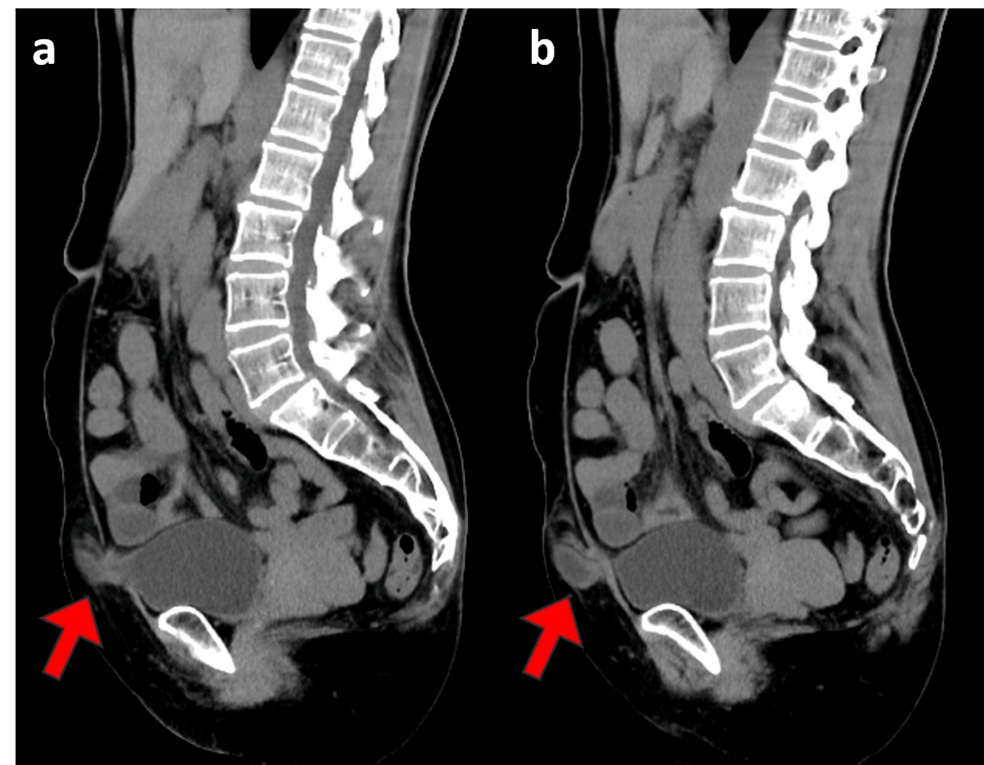
CT scan of the abdomen (sagittal). a, b: The abdominal incisional hernia in the left lower abdomen and a prolapsed bladder from the same area.

We diagnosed an abdominal incisional bladder hernia (Figures 2, 3).

Past history

The patient had two cesarean sections in her 30s. The second cesarean section resulted in a surgical site infection. A high degree of adhesion due to surgical site infection was expected. The plan was to operate using a hybrid technique of small abdominal incision and single-hole TEP. A skin incision of approximately 3 cm was placed directly above the expected hernia portal. Few adhesions were observed. Identification of the hernia portal and prolapsed bladder was easy. We decided to perform surgery using only a small incision. The size of the hernia portal was approximately 1.3 cm. We repaired the hernia portal with direct suture closure.

Operation record

**Figure 4:**
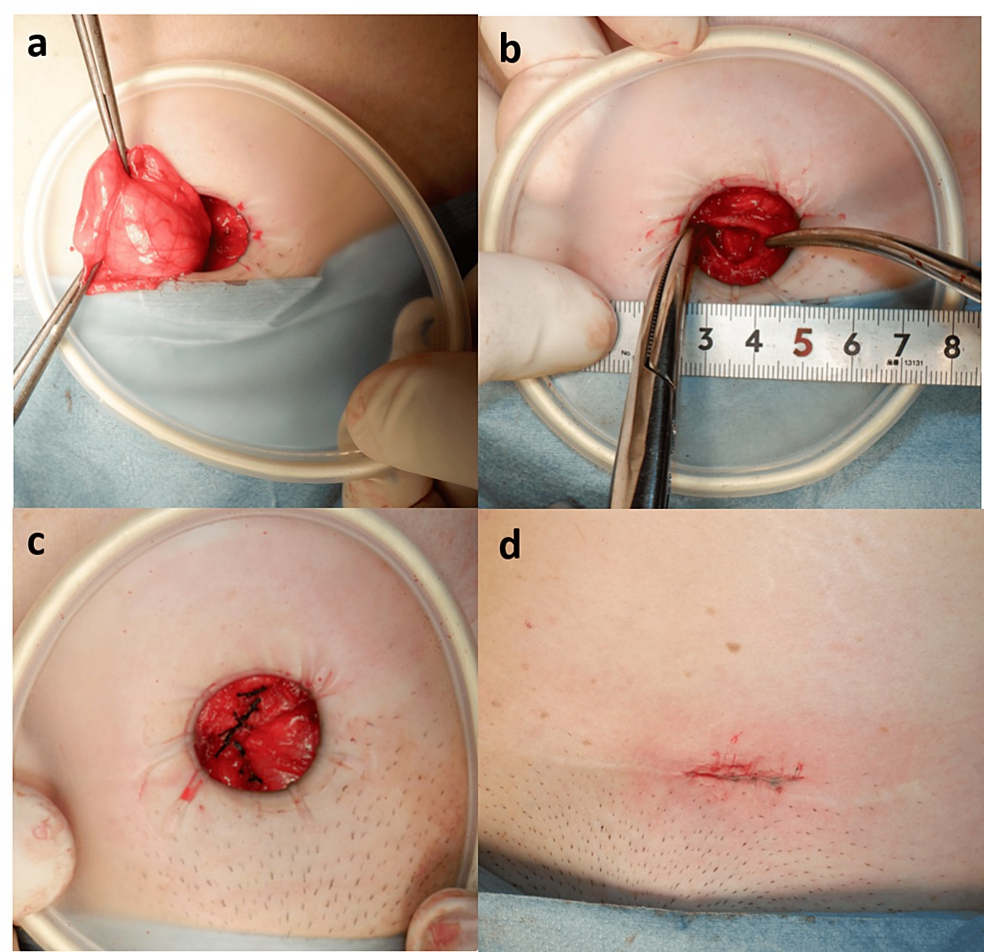
Operative findings. a. A bladder wall prolapse was observed. b. The hernia portal was approximately 1.3 cm. c. Because the hernia portal was 13 mm in diameter, direct suture closure was performed with six stitches of 0 Surgilon. d. A skin incision of approximately 3 cm was placed directly above the hernia portal. Image after closed wound.

A skin incision of approximately 3 cm was placed directly above the expected hernia portal. The patient had an abdominal wall scar hernia due to wound infection after the cesarean section, and preoperative computed tomography (CT) and ultrasound (US) showed scarring around the hernia portal and fistula formation. The plan was to use mesh, but to do so, the fistula had to be dissected dorsal to the rectus abdominal and the bladder dropped completely dorsal to the Retzius cavity, so we were also prepared for a single-hole TEP. The bladder was exposed when the subcutaneous fatty tissue was dissected. Grasping and traction of the bladder revealed a thumb-sized bladder prolapse. We confirmed that there was no difference from the initial preoperative findings. The fatty tissue around the hernia portal and bladder was mildly adherent, and this was sharply and bluntly dissected. Because the hernia portal was 13 mm in diameter, direct suture closure was performed with six stitches of 0 Surgilon. The wound was sutured closed in two layers, and the surgery was completed (Figure 4).

Postoperative course

The postoperative course was good. The patient was discharged on the second postoperative day. It has now been four months since the operation and there has been no recurrence.

## Discussion

Abdominal incisional hernia is a postoperative complication that occurs in 3-11% of abdominal surgeries [1]. It is one of the most common complications. The fascial layer under the surgical wound has separated, and the contents of the abdominal cavity have escaped subcutaneously [2]. The causes are reported to include infection and hematoma formation in surgical wounds, weakening of the abdominal wall due to aging, diabetes mellitus, increased abdominal pressure due to obesity, and the presence of emergency surgery [3]. The hernia contents are mostly small intestine, colon, and large mesentery, and prolapse of the bladder is very rare. PubMed search using the keywords "incisional bladder hernia" revealed eight cases reported from 1985 to 2024 [4-9]. This case was considered rare. There are several symptoms of incisional bladder hernia [8], such as discomfort of the hernia, urinary incontinence, dysuria, urinary frequency, nocturia, hematuria, and two-step micturition. These symptoms may be related to bladder outlet obstruction or secondary to urinary infection often associated with bladder hernia. In our case, the patient complained of lower abdominal distension and pain. The symptoms were particularly enhanced before urination. The increased bladder volume would have increased the hernia content, resulting in an intensification of symptoms.

Common methods for closure include primary closure, open or laparoscopic mesh repair, and the component separation technique. Abdominal incisional hernia repair requires not only hernia repair but also functional and cosmetic repair of the abdominal wall reconstruction. It has been suggested that hernias smaller than 3 cm should be closed primarily with a suture, whereas hernia portals larger than 10 to 15 cm should be closed with a mesh [2,10]. The hernia portal was very small, approximately 1.3 cm in this case. We considered the size of the hernia portal and found the primary suture sufficient. Therefore, we performed a primary suture with nonabsorbable sutures (0 Surgilon used). In previous reports, the use of a mesh has also been considered according to the size of the hernia portal. The operation was completed only by simple closure in a case report with a 1 cm hernia portal as large as the present case [6]. In other cases, meshes have been used in reports of hernia gates larger than 3 cm [7]. In another case, with a large hernia gate of 3.8 cm, ischemia of the bladder and intestinal tract could not be ruled out, and the operation was completed with simple closure alone [8]. We believe that using meshes should be considered for individual case conditions. In conclusion, bladder involvement in hernias other than peri-inguinal hernias is rare. However, it is important to remember that a low abdominal wall hernia, whether in the groin or elsewhere, should be considered to contain bladder tissue until proven otherwise.

## Conclusions

The patient in this case experienced bladder prolapse within an abdominal incisional hernia. The hernia content is rarely a bladder. Preoperative diagnosis can be difficult in some cases, but in others, such as this case, it is possible. In cases with organ prolapse into an abdominal incisional hernia in the lower abdominal wall, we considered it important to list it in the differential, considering urinary symptoms and other symptoms. In this case, it is necessary to select a surgical technique according to the patient's general condition and hernia portal size. Since this is a rare case, long-term follow-up is important to confirm the absence of recurrence, and it is important to continue to accumulate more cases.
